# Supplementary material for: Transcriptome analyses in juvenile yellow perch (Perca flavescens) exposed in vivo to clothianidin and chlorantraniliprole: Possible sampling bias
Source: PLoS One. 2024 Apr 16;19(4):e0302126. doi: 10.1371/journal.pone.0302126 (PMC11020500; doi:10.1371/journal.pone.0302126)
Supplement: S1 File — (DOCX) [file pone.0302126.s001.docx]

**SUPPORTING INFORMATION**

Transcriptome analyses in juvenile yellow perch (*Perca flavescens*) exposed in vivo to clothianidin and chlorantraniliprole: possible sampling bias

Maeva Giraudo^1^, Laurie Mercier^1^, Andrée Gendron^1^, Jim Sherry^2^, Magali Houde^1*^

^1^ Environment and Climate Change Canada, Aquatic Contaminants Research Division, Montreal, Quebec, Canada

^2^ Environment and Climate Change Canada, Aquatic Contaminants Research Division, Burlington, Ontario, Canada

*Corresponding author

Email: [magali.houde@ec.gc.ca](mailto:magali.houde@ec.gc.ca)

### 1. Rainbow trout maintenance and sampling

Juvenile female rainbow trout (n=40) were obtained from Arpents Verts fish farm (Ste-Edwidge, QC, Canada) and maintained following EPS1/RM/9 Environment and Climate Change Canada’s method (Environment Canada 1990) in 400 L polyethylene tanks with flow-through municipal dechlorinated water at 15 ± 2°C under a 16:8 h light:dark photoperiod. Trout (mean mass 0.90g) were then randomly placed in four 30L tanks (n = 10 fish per tank) and maintained in unexposed water for 28 d in the exact same condition than for the yellow perch pesticide exposure. After 28 d, trout from each tank were euthanized in 100 mg/L buffered MS-222 solution in the same chronological order than yellow perch: early morning (E-AM), late morning (L-AM), early afternoon (E-PM), and late afternoon (L-PM) (n = 10 per time point). Fish were measured, weighed (Table S2), and liver were sampled and stored in RNAlater^TM^ stabilization solution (ThermoFisher Scientific, Mississauga, ON, Canada) for gene transcription measurements.

### 2. Quantitative real-time PCR (qRT-PCR)

#### 2.1 Yellow perch RNA-sequencing validation

For each treatment condition (A, CLO, CH, M), total RNA (300 ng) was extracted from individual perch liver (n = 12 per treatment) and was reverse‐transcribed using the QuantiTect® Reverse transcription kit (Qiagen, ON, Canada) per the manufacturer's instructions. The qRT‐PCR analyses were performed on a CFX96 Touch® realtime PCR detection system using SsoAdvanced™ Universal SYBR® Green Supermix (Bio‐Rad, ON, Canada) with a final concentration of 400 nM for each primer in a total reaction volume of 13 μL. The PCR conditions were as follows: 95 °C for 30 s, followed by 40 cycles of 95 °C for 5 s and 60 °C for 5 sec for amplicons <100 bp or 10 sec for amplicons >100bp. Primers for the 8 target genes were designed using PrimerQuest® tool (<https://www.idtdna.com/pages/tools/primerquest>) based on the corresponding transcript sequence from the RNA-seq results. Primer sequences were checked for secondary structures and primer-dimer using OligoAnalyzer (<https://www.idtdna.com/calc/analyzer>) and purchased from Integrated DNA Technologies (Mississauga, ON, Canada). Data were normalized using mRNA levels of 3 reference genes with the most stable transcription level across experiments according to the geNorm algorithm (Vandesompele et al. 2002): *peptidyl-prolyl cis-trans isomerase B* (*pdpi*), *RNA polymerase III subunit F* (*pol3rf*), and *beta-actin* (*actb*). Gene names, symbols, and accession numbers as well as primer‐specific amplification efficiencies, sequences, and lengths of amplification products are detailed in Table S3. Each reaction was run in technical duplicate, and the mean of the 12 independent biological replicates was calculated. Results were analyzed using CFX Maestro^TM^ 1.1 software (Bio-Rad). Relative transcription values were calculated using the comparative threshold method (ΔΔCt; Livak and Schmittgen 2001). Differences in gene transcription levels were tested between exposed and non-exposed fish using one-way ANOVAs followed by Tukey HSD post hoc tests when the means were significantly different from each other. Bonferroni and Benjamini-Hochberg adjustments were used to achieve the family-wise error rate (FWER).

#### 2.2 Transcription analysis of genes related to the circadian rhythm

For each time point (E-AM, L-AM, E-PM, and L-PM), total RNA (300 ng) was extracted from individual trout liver (n=10 per time point) and used as template for reverse-transcription and qRT-PCR following the protocol described for yellow perch (see paragraph 3.1 above). Data were normalized using the two reference genes with the most stable transcription level across experiments according to the geNorm algorithm: *cry1* and *arntl1*. Primer details can be found in S3 Table. Data analyses were performed on the mean of the 10 biological replicates as described in paragraph 3.1 above.
