# Supplementary material for: Transcriptome analyses in juvenile yellow perch (Perca flavescens) exposed in vivo to clothianidin and chlorantraniliprole: Possible sampling bias
Source: PLoS One. 2024 Apr 16;19(4):e0302126. doi: 10.1371/journal.pone.0302126 (PMC11020500; doi:10.1371/journal.pone.0302126)

**S1 Figure.** Heatmap of the top 100 most variable genes in juvenile yellow perch liver exposed to pesticides (A: acetone solvent control, CH: chlorantraniliprole, CLO: clothianidin, M: mixture of both pesticides). Expression values of samples for genes with > 1 CPM in at least 10 samples were ordinated using hierarchical clustering using only the top 100 genes with the largest standard deviations of their log2(CPM) values.


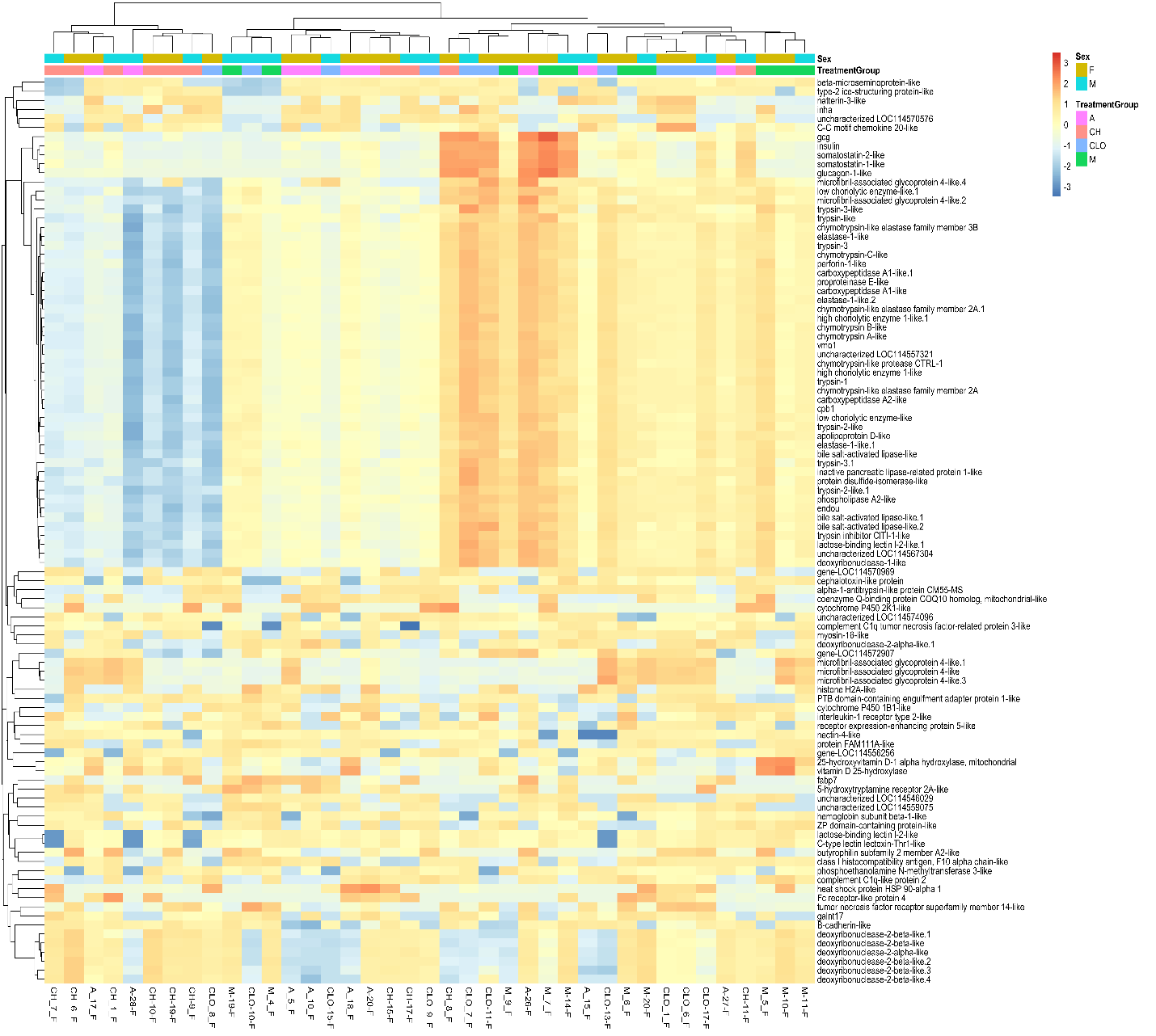

Supplement: S1 Fig — Expression values of samples for genes with > 1 CPM in at least 10 samples were ordinated using hierarchical clustering using only the top 100 genes with the largest standard deviations of their log2(CPM) values. (DOCX) [file pone.0302126.s002.docx]
