# Supplementary material for: Transcriptome analyses in juvenile yellow perch (Perca flavescens) exposed in vivo to clothianidin and chlorantraniliprole: Possible sampling bias
Source: PLoS One. 2024 Apr 16;19(4):e0302126. doi: 10.1371/journal.pone.0302126 (PMC11020500; doi:10.1371/journal.pone.0302126)

**S2 Figure.** Multidimensional scaling (MDS) plot of the filtered genes detected by RNA-seq in yellow perch liver samples (n=37) exposed to pesticides (A: control, CH: chlorantraniliprole, CLO: clothianidin, M: mixture of both pesticides).


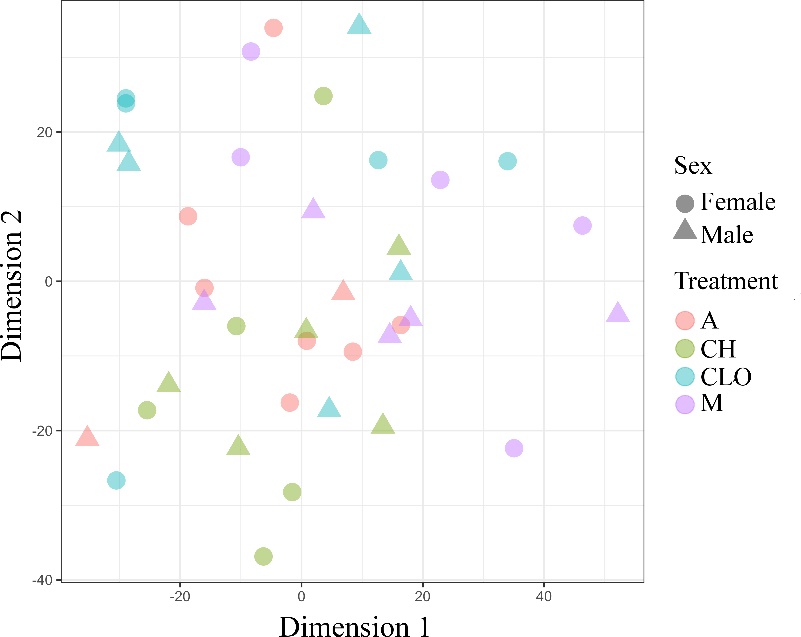

Supplement: S2 Fig — (DOCX) [file pone.0302126.s003.docx]
