# Supplementary material for: Transcriptome analyses in juvenile yellow perch (Perca flavescens) exposed in vivo to clothianidin and chlorantraniliprole: Possible sampling bias
Source: PLoS One. 2024 Apr 16;19(4):e0302126. doi: 10.1371/journal.pone.0302126 (PMC11020500; doi:10.1371/journal.pone.0302126)

**S4 Figure.** Protein-protein interaction network of DTGs in the liver of juvenile YP exposed to a mixture of clothianidin and chlorantraniliprole using the STRING App in Cytoscape. The red and green nodes represent upregulated and downregulated genes, respectively. Zebrafish orthologs of each perch DTG were used as gene list input.


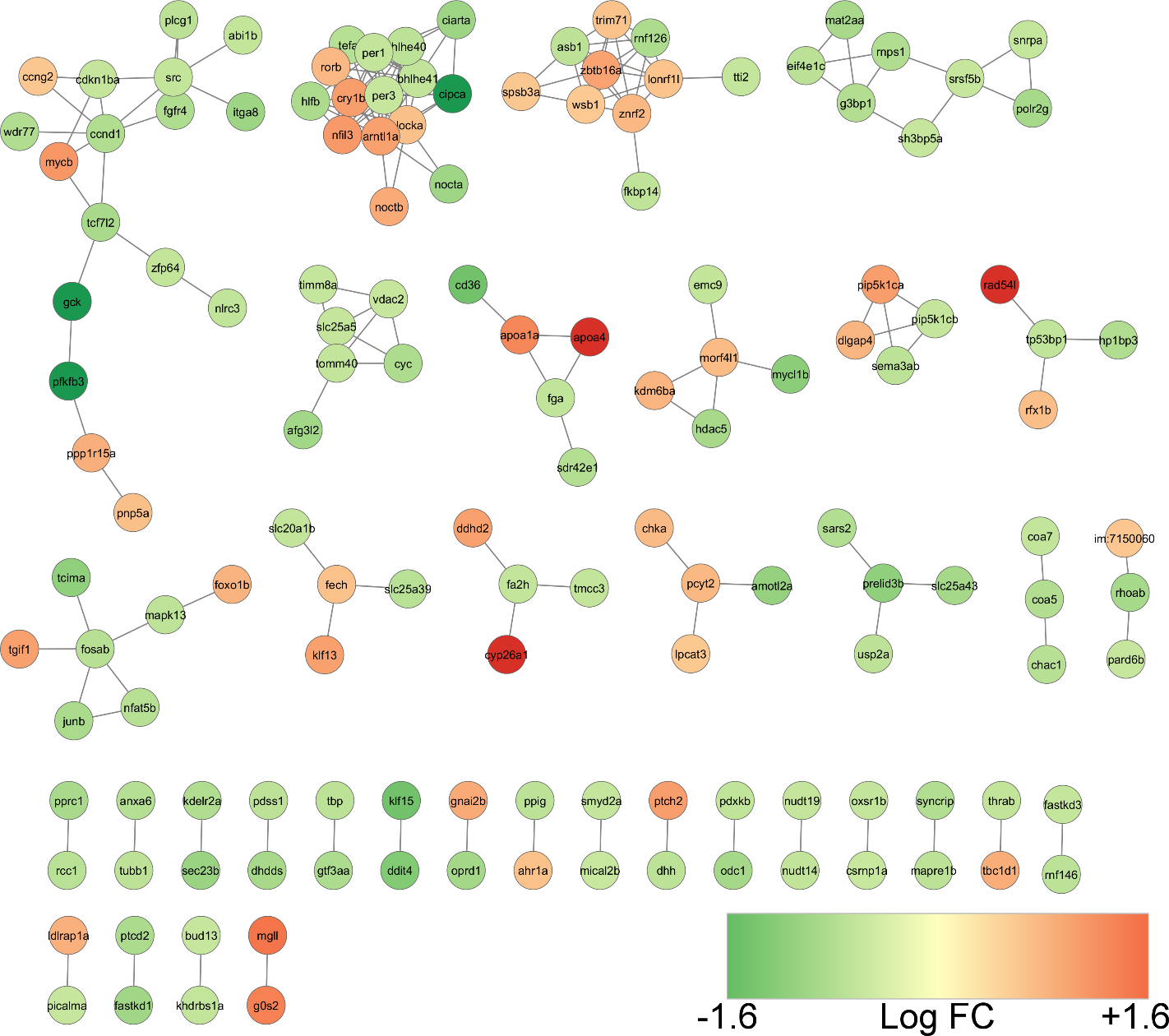

Supplement: S4 Fig — The red and green nodes represent upregulated and downregulated genes, respectively. Zebrafish orthologs of each perch DTG were used as gene list input. (DOCX) [file pone.0302126.s005.docx]
