# Supplementary material for: Transcriptome analyses in juvenile yellow perch (Perca flavescens) exposed in vivo to clothianidin and chlorantraniliprole: Possible sampling bias
Source: PLoS One. 2024 Apr 16;19(4):e0302126. doi: 10.1371/journal.pone.0302126 (PMC11020500; doi:10.1371/journal.pone.0302126)

**S5 Figure.** Correlation between the relative quantification of gene transcription levels measured by qRT-PCR and RNAseq in yellow perch exposed to chlorantraniliprole (CH, blue) and a mixture of CH and clothianidin (M, red). Data are expressed as the Log_2_(fold change) of the mean transcription values between treatment and control conditions (n=12 for qRT-PCR and N=10 for RNAseq).


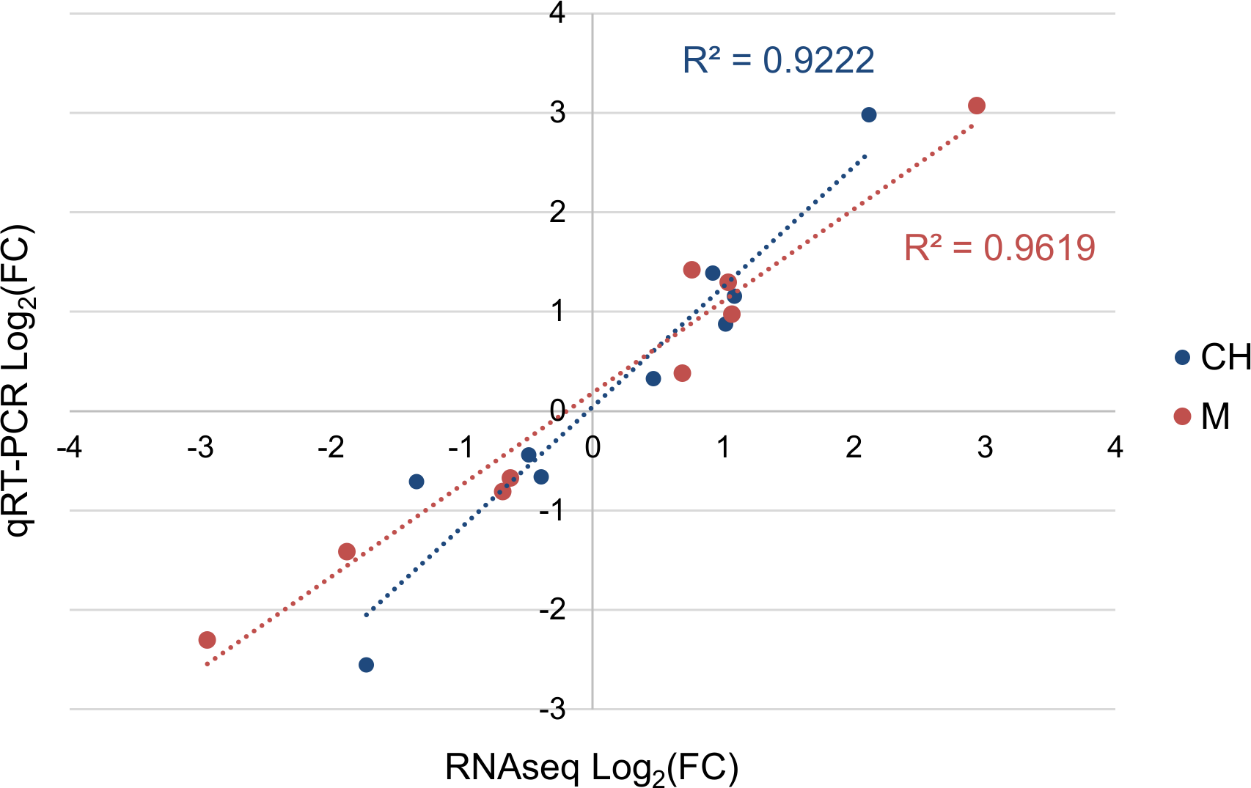

Supplement: S5 Fig — Data are expressed as the Log2(fold change) of the mean transcription values between treatment and control conditions (n = 12 for qRT-PCR and N = 10 for RNAseq). (DOCX) [file pone.0302126.s006.docx]
