# Supplementary material for: Transcriptome analyses in juvenile yellow perch (Perca flavescens) exposed in vivo to clothianidin and chlorantraniliprole: Possible sampling bias
Source: PLoS One. 2024 Apr 16;19(4):e0302126. doi: 10.1371/journal.pone.0302126 (PMC11020500; doi:10.1371/journal.pone.0302126)

**S6 Figure.** Relative gene transcription levels of circadian genes measured by qRT-PCR in yellow perch exposed to pesticides (A) and in rainbow trout sampled at different times of day (B). Data are expressed as the fold change of the mean relative transcription values (n=12 for perch, n=10 for trout). Asterisks indicate a significant difference from the control (A) or from early am sampling time (B).


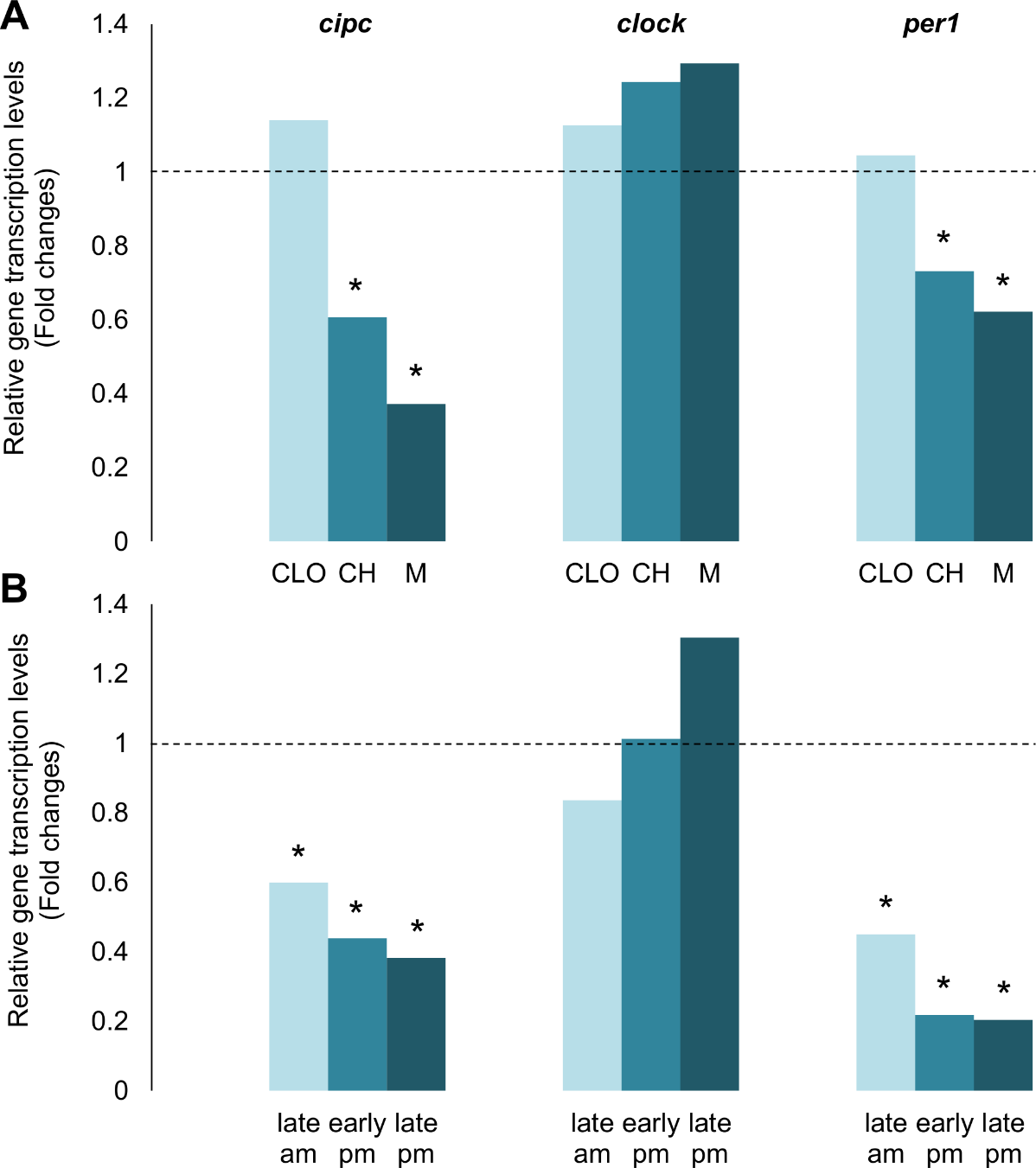

Supplement: S6 Fig — Relative gene transcription levels of circadian genes measured by qRT-PCR in yellow perch exposed to pesticides (A) and in rainbow trout sampled at different times of day (B). Data are expressed as the fold change of the mean relative transcription values (n = 12 for perch, n = 10 for trout). Asterisks indicate a significant difference from the control (A) or from early am sampling time (B). (DOCX) [file pone.0302126.s007.docx]
