# Supplementary material for: Transcriptome analyses in juvenile yellow perch (Perca flavescens) exposed in vivo to clothianidin and chlorantraniliprole: Possible sampling bias
Source: PLoS One. 2024 Apr 16;19(4):e0302126. doi: 10.1371/journal.pone.0302126 (PMC11020500; doi:10.1371/journal.pone.0302126)
Supplement: Attachment — Submitted filename: Responses to Reviewers and Publisher.docx [file pone.0302126.s015.docx]

**Responses to Reviewers and Publisher**

Thank you for the review of our manuscript PONE-D-23-30911 - *Transcriptome analyses in juvenile yellow perch (Perca flavescens) exposed in vivo to clothianidin and chlorantraniliprole: possible sampling bias.* We appreciate the thoughtful comments provided by the two reviewers. Our responses can be found below in blue. Page numbers indicated refer to the track-change revised manuscript.

As part of the revised manuscript submission we have included a track-changes version of the original paper showing all changes made in response to the reviewers comments.

**Responses to reviewers -**

**Reviewer #1:** This manuscript reports a study investigating the effect of clothianidin and chlorantraniliprole on the hepatic gene expression of juvenile yellow perch exposed to each compound alone and in a mixture for 28 days. Novel information regarding circadian rhythm effects in experimental design and results analyses was suggested. Overall, the experiments appear to be robustly performed.

Since the changes in gene expression have been analyzed after exposure for 28 days, therefore, it is likely that these enriched genes may be responsible for the effects related to compensation. Potentially supporting my argument is that the data presented from relative expression levels of circadian genes in Figure S6, and the similar weight and length across all groups (table S2).	**Response:** Although possible, it would be highly speculative to suggest that the observed lack of difference in growth between pesticide-exposed and control fish is the manifestation of a compensatory response to insecticide exposure. This could for instance imply that exposed perch have ingested more food than control fish to compensate for energy loss due to pesticide exposure. Unfortunately, we have no data to support such hypothesis. However, the increase in AChE activity observed after exposure to clothianidin alone and in mixture could indeed be a secondary response of the neurons to the insecticide. Clothianidin is not known to directly influence the activity of AChE; rather, it binds selectively to nicotinic acetylcholine receptors. This binding prevents the inactivation of the receptors by AChE, which results in an overstimulation of the nervous system. In this case, the observed increase in AChE activity is likely a compensatory mechanism induced to degrade the accumulating acetylcholine and thus reestablish the neurons normal activity. We have included this explanation in the Discussion section (L563-565): “*Hence, induction of AChE activity could be a compensatory response mechanism to degrade excess acetylcholine, as has been suggested in zebrafish exposed to 0.87 to 3.51 mg/L sulfoxaflor for 24 to 96 hours.*”

Reviewer #2: The study “Transcriptome analyses in juvenile yellow perch (*Perca flavescens*) exposed in vivo to clothianidin and chlorantraniliprole: possible sampling bias” examined the effects of two insecticides on their own and in combination on juvenile yellow perch liver transcriptomes and brain acetylcholinesterase activity. I would first like to commend the authors on the extent of work that went into the study, and their ability to communicate their findings in a comprehensive and succinct manner.

However, despite finding potential impacts of insecticide impacts on genes associated with calcium signalling, and changes in activity of acetylcholinesterase activity in the brain, a large extent of their significant results appear to be due to differences in sampling times for their treatment groups. I appreciate the effort the authors put into verifying potential impacts of sampling time by examining similar clock genes in rainbow trout, but it does unfortunately highlight an important error in the design of the experiment. If the authors wish to publish the study in its current form, I believe that discussion of the differentially transcribed clock genes needs to be greatly reduced. The connection the authors make between calcium signaling genes and clock genes (page 20) also raises questions regarding whether transcription of calcium signaling genes could also be sensitive to the sampling time. Considering the arguments made by the authors on lines 469-470 – “At this stage, it is therefore impossible to conclude on the transcriptional effect of CH on circadian gene regulation” and that they emphasize that these genes regulate 10-20% of the expressed genome (lines 471-472), it becomes difficult to discern what this study offers with respect to our knowledge on the effects of the studied insecticides on the yellow perch liver transcriptome.
**Response:** We understand the reviewer’s point and agree with the suggestion. The text from the sub-section *Functional analyses of differentially transcribed genes* was revised and a large part of the discussion on the differentially transcribed clock genes was deleted (e.g. L444-451, L460-495).

Minor comments:
Lines 269-276 – can the authors provide further justification as to why juvenile rainbow trout were an acceptable alternative to yellow perch? This point may be mute, given the end result. **Response:** Explanation on the reason for using rainbow trout is given on L270 - *mRNA levels of circadian gene orthologs were also measured by qRT-PCR in unexposed juvenile rainbow trout (Oncorhynchus mykiss) due to the lack of YP stock availability outside of the annual breeding period.* Additional information to justify the use of rainbow trout was added to the text on L279: *Both fish species are teleosts and the controlled breeding of female rainbow trout in our laboratory allowed us to reproduce the exposure conditions of perch regarding size, weight, and carrying capacity. The sequencing of trout genome also facilitated the qRT-PCR analysis.*

Line 307-308 – were differences in weight and length among treatment groups analyzed statistically?
**Response:** Yes. The results of the statistical comparison among treatment groups for fish length and weight have been added on L316-317.

Line 314 – Can the authors include their reasoning on why a minimum of 1 CPM in at least ten samples was chosen as the threshold for DTG filtering.	**Response:** It is recommended to filter out lowly expressed genes because very low counts across all samples provide little evidence for differential expression and because they add to the multiple testing burden when estimating false discovery rates, reducing the statistical power to detect differentially expressed genes. As a rule of thumb, the filtering threshold is chosen by identifying the CPM (counts per million) that corresponds to an actual count of 10 for the library sizes, which in our dataset is 1. CPMs are used rather than actual counts, as the latter does not account for differences in library sizes between samples. The CPM threshold is required in at least 10 samples because this is the sample size for the groups in our experiment.	This text has been added on L323-329: *Because very low counts across all samples provide little evidence for differential expression and because they add to the multiple testing burden when estimating false discovery rates, lowly expressed genes that did not reach a minimum of 1 count per millions reads (CPM) in at least ten samples were filtered out. This CPM value corresponds to a minimum of 10 counts in our librairies, which is the recommended threshold (Chen et al 2023).*

Can the authors elaborate on how the comparison was made between the CLO+CH and M effects on differentially transcribed genes.	**Response:** Contrasts analyses were done to compare CLO+CH and M effects. Contrasts are specified using the corresponding linear model formula. For instance, B-A refers to the comparison of group B to A, using A as the denominator in the comparison. FC were therefore calculated by dividing the transcription values of each condition by the common denominator A (control): CLO/A, CH/A, M/A. For the interaction, the test verifies that exposure to the mixture differs from the sum of the effects of the 2 pesticides taken individually: (M+A)/(CH+CLO).

**Responses to the publisher –**

1. Please ensure that your manuscript meets PLOS ONE's style requirements, including those for file naming.	**Response:** The manuscript has been revised based on the two template documents.

2. We note that the grant information you provided in the ‘Funding Information’ and ‘Financial Disclosure’ sections do not match. When you resubmit, please ensure that you provide the correct grant numbers for the awards you received for your study in the ‘Funding Information’ section. **Response:** Funding information has been added in the system. No grant number is associated with this funding.

3. Thank you for stating the following in the Acknowledgments Section of your manuscript. We note that you have provided funding information that is currently declared in your Funding Statement. However, funding information should not appear in the Acknowledgments section or other areas of your manuscript. Please remove any funding-related text from the manuscript and let us know how you would like to update your Funding Statement. Please include your amended statements within your cover letter; we will change the online submission form on your behalf.	**Response:** The funding information has been deleted from the Acknowledgement Section of the manuscript.

4. PLOS requires an ORCID iD for the corresponding author in Editorial Manager on papers submitted after December 6th, 2016. Please ensure that you have an ORCID iD and that it is validated in Editorial Manager.	**Response:** The ORCID information has been updated in the corresponding author profile and added in the submission system.
