# Supplementary material for: Transcriptome analyses in juvenile yellow perch (Perca flavescens) exposed in vivo to clothianidin and chlorantraniliprole: Possible sampling bias
Source: PLoS One. 2024 Apr 16;19(4):e0302126. doi: 10.1371/journal.pone.0302126 (PMC11020500; doi:10.1371/journal.pone.0302126)
Supplement: Attachment — Submitted filename: Responses to Reviewer #3.docx [file pone.0302126.s016.docx]

**Responses to Reviewers**

This reviewer was not part of the original review and has been brought on board to evaluate the first revision. The foundation of the data used to elucidate the molecular mechanisms of the pesticides is based on RNA sequencing and qRTPCR. The experiments were well designed and performed and were not criticized in the first review, this reviewer has some concerns about the details of the quantitative transcriptomics experiment. These concerns are not likely to be fatal however, they should be addressed.

The authors are referred to section 7.4.1. PCR efficiency in the following document, provided with the review - Bustin et al. 2009 MIQE Minimum Information for Publication of Quantitative Real-Time PCR. Clinical Chemistry 55:4 611–622.

**Responses to the reviewer**

As requested by the reviewer we have added information on the efficiency of the PCR analyses. This sentence has been added on L275 - *Percent efficiencies (90-110) and the coefficient of determination (r2>0.980) of calibration curves respected the standards recommended in Taylor et al (46*). The reference has also been added to the list.
